# Vegetarianísh—How “Flexitarian” Eating Patterns Are Defined and Their Role in Global Food-Based Dietary Guidance

**DOI:** 10.3390/nu17142369

**Published:** 2025-07-19

**Authors:** Julie M. Hess, Kaden Robinson, Angela J. Scheett

**Affiliations:** 1 US Department of Agriculture, Agricultural Research Service, Grand Forks Human Nutrition Research Center, Grand Forks, ND 58203, USA; 2 Department of Nutrition & Dietetics, University of North Dakota, Grand Forks, ND 58202, USA

**Keywords:** flexitarian, vegetarian, plant-based, dietary guidance, food-based dietary guidance, global

## Abstract

**Background/Objectives:** A dietary pattern that simply reduces animal-based foods may be more acceptable to consumers than strict vegetarian or vegan diets. The objective of this investigation was to identify the most consistently used definitions of “flexitarian” dietary patterns, or dietary patterns with a reduced amount of animal foods. Then, sets of food-based dietary guidance (FBDG) from different countries and regions were evaluated to determine whether their guidance could accommodate flexitarian diets. **Methods:** Literature searches yielded 86 total results on flexitarian eating after screening by title/abstract, full text availability, and English language. Definitions of “flexitarian” were extracted from each article then reviewed and summarized. FBDGs available in English were downloaded from the Food and Agriculture Organization of the United Nations website. Guidance related to reduced animal product diets was extracted from FBDGs for eating patterns closest to 2000 kcal. **Results:** The summary definition of flexitarian included eating at least one animal product (dairy, eggs, meat, or fish) at least once per month but less than once per week. FBDGs from n = 42 countries or regions were downloaded and data extracted. Only FBDG from Sri Lanka explicitly describe a “semi-vegetarian” eating pattern, though n = 12 FBDGs describe a vegetarian pattern and n = 14 recommend reducing meat or animal food and/or choosing meat/dairy alternatives. **Conclusions:** Following a flexitarian dietary pattern in terms of reducing or limiting red meat is feasible and even implicitly recommended by the official dietary guidance of several countries. Most FBDGs examined did not include recommendations to decrease dairy or fish intake.

## 1. Introduction

Despite a burgeoning interest in plant-based eating, few people adopt restrictive plant-based eating patterns such as vegetarian or vegan diets [1]. Consumer insights research indicates that, in 2024, more Americans reported following eating patterns with reduced animal foods than more strictly defined vegetarian or vegan diets [2]. While only 2% of consumers indicate that they follow vegan diets and 3% of consumers report following a vegetarian diet, 5% of American consumers followed “flexitarian” diets [2]. The reasons for following more plant-oriented dietary patterns varies, though most consumers (55%) follow these patterns to be healthier, because they enjoy plant-foods more (38%), or to improve animal welfare (33%) [2].

Including plant-based foods and reducing animal-based foods in the diet has also been a common topic in food based dietary guidance (FBDG) that addresses sustainability as a basis for diet recommendations [3]. The shift to recommending plant-based diets as an environmental mitigation strategy happened within the span of about 10 years. Several countries have introduced environmental sustainability into their FBDGs since 2010, when the Food and Agriculture Organization of the United Nations (FAO) released a report entitled “Sustainable diets and Biodiversity” [4]. This report noted that dietary changes were not frequently discussed in the climate change literature, and it recommended the global development and adoption of “sustainable diets” [4]. Accordingly, by 2016, an updated FAO report indicated that four countries explicitly discussed environmental sustainability in their official FBDGs (Brazil, Qatar, Sweden, and Germany) [5]. When a review of the inclusion of environmental sustainability in dietary guidance was published in 2022 [3], 37 countries with guidance translatable into English mentioned environmental sustainability out of 83 total sets of FBDGs. Most of these countries are located in Europe and Central Asia or Latin America and the Caribbean [3].

“Flexitarian” dietary patterns have been defined as a potential strategy for consumers to reduce their animal food intake without removing it completely. Broadly, the term “flexitarian” describes dietary patterns with animal-source food intake somewhere between vegan or vegetarian diets and omnivorous diets. This term does not have a specific definition or quantifiable properties and, therefore, can apply to dietary patterns with a variety of animal intakes. Because there is no formal or even colloquial definition, “flexitarian” patterns are primarily self-defined. There are several terms similar to “flexitarian” also utilized in the nutrition science literature, including “semi-vegetarian,” “flexible vegetarian,” “meat-reducers,” and “reducetarian,” among others [6,7,8].

Despite the interest in plant-based eating that still includes some animal-source foods [9], relatively little research has been dedicated to defining flexitarian dietary patterns, especially patterns that include lower amounts of animal-sourced foods but still meet nutrient needs for average consumers. The term “flexitarian” was mentioned in the popular press in the early 2000s [10] but did not become a mainstream term until 2010 when registered dietitian Dawn Jackson Blatner published *The Flexitarian Diet: The Most Vegetarian Way to Lose Weight, Be Healthier, Prevent Disease, and Add Years to Your Life* [11]. Blatner describes the diet as mostly plant-based food but with the flexibility to include meat occasionally [11]. She also describes flexitarian eating as a “casual vegetarian” diet, identifying three “levels” of adherence [11]. Beginner flexitarians have two meatless days per week, advanced flexitarians limit meat intake to three or four days a week, and expert flexitarians avoid meat on five out of seven days per week. An early use of the term “flexitarian” in the peer-reviewed literature mentions Blatner’s role in identifying this unique dietary pattern and the importance of differentiating between individuals who eat meat frequently and those who eat meat occasionally, noting that prior studies have largely ignored the flexitarian subgroup and/or subsumed them into a larger group with omnivores [12].

Our hypothesis was that, to date, there is not a commonly used parameter specifying the amount of animal foods in “flexitarian” diets. Furthermore, we hypothesized that FBDGs do not explicitly use the terms “flexitarian,” “semi-vegetarian,” or “reducetarian” to describe recommended diets. The objectives of this study were twofold:

1. Identify the most used definitions of “flexitarian” dietary patterns in the scientific literature and develop draft quantitative parameters;
2. Assess dietary guidance available in English to see how well guidance from the food-based dietary guidance (FBDG) of different countries aligns with most common definitions of “flexitarian” in the literature.

## 2. Materials and Methods

### 2.1. Defining Flexitarian Diets

**Figure 1:**
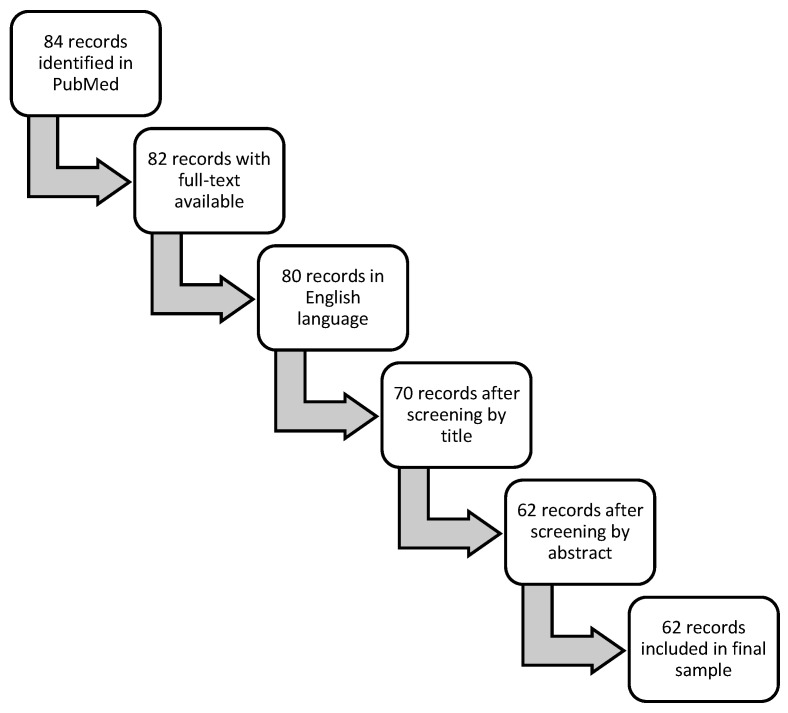
Flow diagram of records selection process for articles defining “flexitarian” among the scientific literature published in PubMed.

A PubMed search in September 2023 of the term “flexitarian” yielded 84 manuscripts, and n = 62 of these manuscripts were included in the final review (Supplementary Materials). Only full-text articles available in the English language that were relevant to this topic as determined by title and abstract review were included. Figure 1 provides an overview of the search structure. This search protocol was not registered.

**Figure 2:**
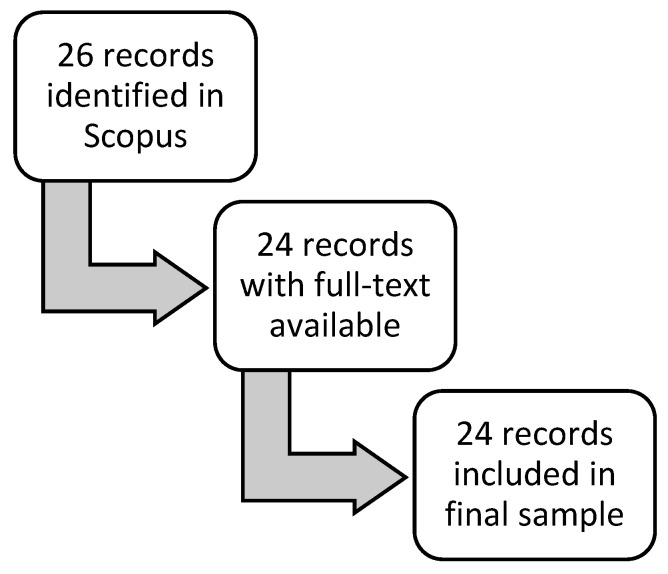
Flow diagram of records selection process for articles defining “flexitarian” among the scientific literature published in Scopus.

A second search was conducted with Scopus in June 2025 to find additional literature that may have been published prior to September 2023 but was not captured in the original PubMed search. The full search strategy can be found in the Supplementary Materials. Figure 2 provides a graphical illustration of this secondary literature search.

### 2.2. Finding FBDGs to Include 

The FAO maintains a webpage with links to FBDGs available in 100 countries [13]. This webpage was utilized to identify countries with dietary guidance available in English, a strategy that has been employed in previous research efforts [3]. A copy of each set of FBDG listed on the webpage that was available in English was downloaded and documented.

Data was extracted from each file, including country name, year of dietary guidance, sex/age recommendations for each dietary pattern including approximately 2000 kcal, and servings of each of nine food groups (vegetables, fruits, grains, dairy, meat and eggs, seafood/fish, legumes/pulses, nuts/seeds, and oils/fats) within a recommended dietary pattern closest to 2000 kcal. For instance, Afghani dietary guidance had patterns at 1300, 2200, and 2800 kcal, so data on the 2200 kcal pattern was extracted for this analysis [14]. One member of the research team conducted the initial data extraction, and a second re-searcher reviewed each entry for accuracy and completion. Any disputes between the first and second researchers were resolved after review by a third researcher.

With each set of FBDGs that recommended multiple eating patterns or styles, the most common or “base” pattern was used in this analysis. For example, the 2020–2025 Dietary Guidelines for Americans (2020 DGA) included Healthy U.S.-Style, Healthy Vegetarian, and Healthy Mediterranean Dietary Patterns [15], so this analysis used the Healthy U.S.-Style Dietary Pattern for comparisons. Guidelines germane to flexitarian-style diets were noted (e.g., eat small amounts of animal foods, mention vegetarian diets as an option, replace meat with alternates), as was flexibility within the guidelines to follow a flexitarian dietary pattern as defined by the literature identified in the PubMed and Scopus searches. Two sets of guidelines provided recommendations for an entire geographic region rather than a specific country (e.g., the Nordic Nutrition guidance [16] and the Pacific Guidelines for Healthy Living [17]). Although these sets of guidance were included in this analysis, throughout this manuscript, FBDGs are described as “country-specific.”

## 3. Results

### 3.1. Literature Review

Data extracted from the n = 86 final studies include the term(s) used within them for dietary patterns with fewer animal products and the definition of those dietary patterns (Table 1). The most common two terms used by these studies for diets with few animal foods were “flexitarian (n = 58) or “semi-vegetarian” (n = 47), with fewer studies using the term “meat-reducers” (n = 9), and only one study each using the terms “reducetarian,” “flexible vegetarian,” “low meat-eater,” “demi-vegetarian,” “casual vegetarians,” “vegivores,” or “flexi-semi-vegetarian.”

Synthesizing the descriptions and definitions of dietary patterns lower in animal products (that did not remove animal food categories completely), there were few specific quantitative descriptions provided. Part of the benefit of a “flexitarian” diet over a strict vegetarian or vegan diet comes from its inherent flexibility. To provide structure for analyzing global FBDGs, the below four items summarize the limits on animal foods mentioned by some of the studies and will be used to identify FBDG that can accommodate a flexitarian diet:

1. Consume dairy products at least once per month but less than once per week;
2. Consume eggs at least once per month but less than once per week;
3. Consume meat and/or poultry products at least once per month but less than once per week;
4. Consume fish and/or seafood at least once per month but less than once per week.

**Figure 3:**
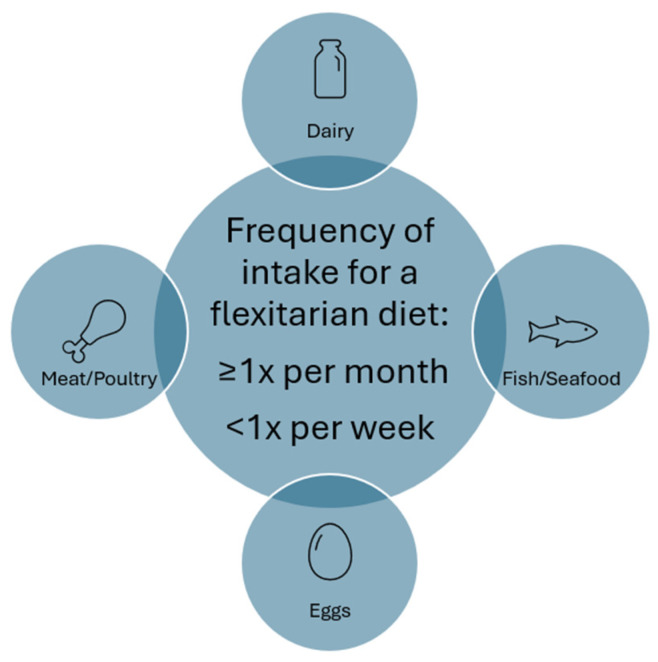
Summary of intake frequency of animal foods within a flexitarian diet as defined by literature review in PubMed and Scopus.

Figure 3 provides a graphical illustration of this summary definition.

### 3.2. Flexitarian Diets in FBDGs

A total of n = 52 FBDGs contained guidance in English and were downloaded for data extraction. Some FBDGs did not contain quantitative guidance or recommendations for specific dietary patterns. These guidelines, which included FBDG from Antigua and Barbuda, Bahamas, Brazil, Canada, Dominica, Guyana, Namibia, Nigeria, Saint Lucia, and Thailand, were removed from consideration. Cambodia’s guidelines [99] were developed specifically for school-aged children and China’s most recent 2022 guidelines were not available for download, so these FBDGs were also excluded.

Fiji published country-specific guidelines in 2013 [100], and new guidelines were published for the Pacific Community in 2018 [17]. The Pacific Community encompasses 27 member countries and territories, including American Samoa, Australia, Cook Islands, Federated States of Micronesia, Fiji, France, French Polynesia, Guam, Kiribati, Marshall Islands, Nauru, New Caledonia, New Zealand, Niue, Northern Mariana Islands, Palau, Papua New Guinea, Pitcairn Islands, Samoa, Solomon Islands, Tokelau, Tonga, Tuvalu, United Kingdom, U.S., Vanuatu, and Wallis and Futuna as well as Fiji. Both sets of these guidelines were included, as it was not clear whether the Pacific Community guidelines was intended to replace the 2013 guidelines specific to Fiji. The total number of FBDGs included in this analysis was n = 42.

None of the FBDGs explicitly named a “flexitarian” eating style; however, dietary guidance from Sri Lanka described a “semi-vegetarian” dietary pattern as part of a list of “Types of Vegetarian Diets” [101]. This pattern was defined as a “mainly plant based diet” that “may include fish/egg/poultry, milk and milk products occasionally or in small quantities” [101]. Several other FBDGs explicitly mentioned vegetarian patterns (n = 12) or reducing meat or animal food and/or choosing meat/dairy alternatives (n = 14). Spanish guidelines from 2022 recommended limiting meat consumption to “a maximum of 3 servings of meat per week” [102]. German guidelines (2024) also recommended limiting intake of meat and sausage and emphasized choosing a diet that is 34 plant-based and 14 animal-based, indicating the ability to follow a flexitarian dietary pattern within their recommendations [103]. Sri Lankan guidelines recommended 23 of protein sources come from plants and 13 of protein sources come from animals [101].

FBDGs (n = 28) from many countries indicated the possibility to follow recommendations within flexitarian definitions with some animal products. For instance, Saudi Arabia’s guidelines [104] recommended that dairy foods be consumed daily and do not provide a quantitative recommendation for egg consumption. These guidelines recommended that meat and substitutes (cooked lean meat, poultry, fish, egg, dry beans, peanut butter) be consumed daily, meaning that beans or legumes could be consumed in lieu of meat and fish. Therefore, it would be feasible to follow a flexitarian dietary pattern following these guidelines by consuming meat, poultry, fish, and seafood less than once per week but more than once per month even though not explicitly recommended. However, a flexitarian approach could not be adopted with dairy foods within the parameters of these guidelines [104]. Flexitarian diets may also be possible with a similar approach using guidance from Afghanistan [14], Australia [105], Belgium [106], Bulgaria [107], Barbados [108], England [109], Fiji [100], Georgia [110], Ghana [111], Ireland [112], Israel [113], Oman [114], Japan [115], Kenya [116], Lebanon [117], the Netherlands [118], New Zealand [119], Nordic countries [16], Norway [120], the Pacific Community [17], the Philippines [121], Qatar [122], Sierra Leone [123], Sweden [124], and Zambia [125]. Diet pattern examples in India’s 2024 guidance [126] focus on vegetarian diets, which meet the definition for flexitarian diets with respect to meat/poultry, fish/seafood, and eggs.

Reducing animal-based food intake was not feasible with other sets of FBDG. Albanian guidance recommended daily consumption of one portion of meat or fish as well as an egg or a portion of cheese in addition to three portions of other dairy foods (milk or yogurt) daily [127]. Similarly, a flexitarian diet would not be possible following guidance from Bangladesh [128] or Ethiopia [129], as both countries recommended daily consumption of dairy as well as poultry, meat, fish, or eggs.

Following a flexitarian dietary pattern may be feasible within the parameters of FBDGs from Jamaica, Saint Kitts and Nevis, St. Vincent and the Grenadines, South Africa, Seychelles, and Belize, but would require reducing intake of one or two animal-based foods and consuming more of other animal-based foods. Jamaica recommended daily intake from an animal food source [130], as did guidance from Saint Kitts and Nevis [131] and St. Vincent and the Grenadines [132]. These guidelines grouped all animal foods (dairy, eggs, meat, and fish) into a single category and recommended consuming 4–8 daily servings from this category. South Africa’s Food-Based Dietary Guidelines [133] also stated that “fish, chicken, lean meat and eggs can be eaten daily” but established weekly limits on each of these categories (no more than 560 g red meat, 3–4 eggs, or 2–3 portions of fish). Some countries such as the Seychelles [134] did not have quantitative recommendations for eggs or meat but recommended three daily servings of dairy and five weekly servings of fish. It would be similarly difficult to follow a flexitarian diet using recommendations from Belize [135], which recommended seven small daily portions of meat, eggs, or seafood/fish.

An overview of the dietary guidance reviewed, and a summary of their quantitative animal food recommendations, can be found in Table 2.

## 4. Discussion

Use of the term “semi-vegetarian” in the literature nearly as frequently as the term “flexitarian” as well as its use in Sri Lankan FBDG indicates that there may be broader acceptance of that term in the scientific literature than “flexitarian.” However, there is still little known or understood about the vegetarian and vegan population, much less how much of the population chooses to define their diets as lower in animal foods as opposed to being strict vegetarians or vegans [138]. Consumers may sometimes or often choose vegetarian meals without defining their dietary pattern in a specific way. Choosing to reduce meat or other animal foods on one day of the week as with “Meatless Mondays” [139] and similar efforts [25] does not meet the definitions used in this analysis for a flexitarian diet but, at least for U.S. consumers, might still decrease meat intake from estimated intake of 104 g/day based on previous analyses from the National Health and Nutrition Examination Survey [140].

Some version of a flexitarian dietary pattern reduced in meat or poultry foods aligns with dietary guidance in several countries; however, few countries have guidance that allows for a flexitarian dietary pattern with limited dairy intake. Many countries recommend daily intake of dairy foods [141]. Some countries (Barbados, Belize, Fiji, Ghana, Jamaica, Pacific Community, Saint Kitts and Nevis, St. Vincent and the Grenadines, and Sierra Leone) in this study recommend consuming dairy foods as part of a broader “animal foods” group including meat, eggs, poultry, and sometimes fish and do not include a specific “dairy foods” group. Each of these countries still recommends daily intake of the animal foods group. England’s Eatwell Guide includes a section of dairy and alternatives (milk, cheese, yogurt, and soya drink) but does not specify that these foods need to be consumed with a specific frequency [109].

Notably, the dairy groups of some countries do also include some plant-based alternatives that provide similar nutrients to milk, cheese, yogurt, and other nutrient-dense dairy foods. Australia and Oman recommend fortified plant-based drinks (soy, rice, oat), Lebanon recommends calcium-fortified soy milk or orange juice, and New Zealand and the Nordic Nutrition Recommendations list fortified plant-based milk alternatives like soy, rice, oat, and nut milk as nutritional equivalents for dairy foods in the diet. Fiji and Israel note soy milk that is unsweetened and, in the case of Israel’s guidelines, free of additives, can be an alternative beverage [100,113]. Qatar includes fortified soy and almond drinks as dairy alternatives, England lists unsweetened and fortified soy milk as an option, and the U.S. is unique in listing both fortified soy milk and soy yogurt as part of its dairy group [15,109,122]. Other countries include food sources of calcium and vitamin D as dairy alternatives instead of beverages. Georgia’s guidelines recommend that people who cannot drink milk choose to eat more dark green vegetables and nutrient-dense grains while Lebanon recommends fortified cereals, and Qatar lists chickpeas as alternate sources of calcium and vitamin D. Sweden recommends sardines as well as plant-based alternative beverages, nuts, and leafy greens as options to provide some of the nutrients found in dairy foods.

Limiting intake of red meat, poultry, and sometimes eggs to a certain number of servings per week was a much more frequent recommendation in the sets of dietary guidance analyzed. Reducing intake of one or both foods may be a more acceptable entry to flexitarian dietary patterns than reducing dairy foods. Germany, Bulgaria, Israel, Sweden, the Netherlands, Norway, Qatar, Spain, England, and South Africa provide quantitative limits for red meat consumption in their guidelines, amounts ranging from <70 g/day of red meat (England) to <350 g of meat per week (Nordic recommendations) to South Africa’s <560 g per week or <90 g per day of red meat. The U.S. recommends 26 ounce-equivalents (oz-eq) of meats, poultry, and eggs weekly in the Healthy U.S.-Style Dietary Pattern, which when divided into 5 to 6.5 oz-eq for daily consumption, amounts to eating meat or poultry about four times per week [15]. India recommends consuming fewer than three eggs per week, Bulgaria recommends up to three servings per week of meat and eggs, and Spain recommends two to four eggs per week. In contrast, Belgium recommends choosing eggs, legumes, fish, or poultry as substitutes for red meat, and Kenyan guidelines suggest that consumers aim to eat meat, eggs, seafood, and fish at least twice per week. Some guidelines (Zambia and Kenya) have animal foods groups that include insects [116,125] and/or caterpillars [125], which were widely discussed as potential options for less environmentally impactful sources of protein in the late 2010s [142,143]. Few locations frame recommendations for animal-source food in terms of minimum intake. A few exceptions include Kenyan guidance that recommends eating “lean meat, fish and seafood, poultry, insects or eggs at least twice a week” and the Seychelles guidance that recommends eating fish “at least 5 days” per week [116,134].

Several countries (Belgium, Bulgaria, England, Germany, Israel, Lebanon, the Netherlands, Nordic, Norway, Oman, Qatar, Seychelles, South Africa, Spain, Sweden, Turkey, the U.S., and Zambia) explicitly recommend eating one or more servings of fish on a weekly basis. Some of these countries (Sweden, Nordic, Norway, Lebanon, England, Zambia) further specify that at least one weekly serving of fish should come from an oily/fatty fish like salmon or herring. There could be multiple reasons for the limits and recommendations on meat, eggs, and fish/seafood intake, depending on the country and regional context. Newer guidelines, such as Zambia’s guidance from 2021, reference the Global Burden of Disease study as presented in the EAT-Lancet report [144]. This report proposes guidance for diets that support both human and planetary health with recommendations intended to be flexible for different cultural traditions and food availability and accessibility. The 2019 EAT-Lancet report proposed global dietary recommendations include reducing animal food intake for environmental reasons, listing ranges for protein sources that start at 0 (zero) for beef and lamb, pork, poultry, eggs, fish, and most legumes as well [144].

## 5. Limitations

FBDGs may have been updated in some countries since the writing of this manuscript. An initial list of countries and their guidance was gathered in spring 2024. In March 2025, a search of all guidelines published prior to 2020 was conducted to ensure no newer guidance was missed. India and Oman’s 2024 guidelines were among those collected in the more recent search. In addition, it was noted that English language versions of all FBDGs were not readily available for download online, including guidance from Malaysia and China. Turkey was scheduled to have new guidance published in 2014 per the FAO WHO website, but a new link has not been provided, so the 2006 version was used for this analysis.

In addition, it was not always clear how some guidance may have been translated to the English language. Some of the subtleties of recommendations may have been lost in the technical translation process. For instance, Albanian guidance for adults recommends a portion of meat, fish, or eggs be consumed daily. However, earlier in the document, meat is described as part of a food group that also includes cooked peas, peanut butter, kidney beans, and nuts. It is not clear whether the recommendation for adults to consume meat, eggs, and fish could also incorporate these vegetarian protein sources [127]. This analysis may have missed subtleties in recommendations from different sets of FBDGs, too, as it is not always clear how the guidance is intended to be applied. Food group recommendations can be reported in a summary table, separate tables, or simply mentioned in the text, and it is not always clear which guidance should take precedent when there are slight differences among them.

Because information on specific dietary parameters (e.g., number of servings of specific animal-source foods) and recommendations often required perusal of entire FBDG reports, it was not possible to utilize FBDGs unavailable in English in this analysis due to the researchers’ lack of fluency in other languages. This necessarily limits the interpretation of the results as they are not entirely representative of all countries that release FBDGs. According to the FAO, approximately 100 countries have FBDGs, and a previous analysis found FBDG for 90 countries [13,145]; therefore, this analysis with only 42 countries represents < 50% of available FBDGs.

As noted earlier, some guidance does not use portions or food groups, and these FBDGs, including those from Canada (2019) and Brazil (2015), were not included in this analysis. Canada’s 2019 guidance largely does not include portions or food groups in their recommendations [146] with the exceptions of vegetables and fruits, protein foods, and whole-grain foods [147]. While not included in this analysis, as a working link was not available on the FAO webpage, this approach to dietary guidance that does not include specific servings and recommendations was also utilized by Brazil in 2015 [148].

The definitions used to establish the parameters of flexitarian diets for this study may be more prescriptive than the term is meant to convey. A 2021 review of flexitarian diet studies noted that variable definitions are inherent to the concept of flexitarianism [46]. A search of only two databases (PubMed and Scopus) for articles that include the term “flexitarian” will have missed journals not indexed in those databases as well as articles that address reduced meat, fish, or dairy diets that, in effect, also discuss flexitarian eating patterns. Finally, the search protocols were not pre-registered, which could have introduced additional bias into the literature search, further limiting the applicability of these findings.

## 6. Conclusions

Following a flexitarian dietary pattern in terms of reducing or limiting red meat is feasible and even implicitly recommended by the official dietary guidance of several countries. However, only one country (Sri Lanka) refers to this reduced animal product diet as “semi-vegetarian,” and no countries utilize the term “flexitarian” in their guidance.

## Figures and Tables

**Table 1:** Descriptions and terminology used for dietary patterns containing small amounts of animal foods in the scientific literature by geographical location.

Geographical Location of Study [Citation]	Term(s) Used for “Flexitarian” Dietary Patterns	How Dietary Pattern Low in Animal Products Are Described/Defined
Australia [18]	Semi-vegetarian	“Individuals who follow a vegetarian diet but occasionally eat meat or poultry”
Australia [19]	Semi-vegetarian	“very minimal and/or infrequent consumption of meat.”
Australia [20]	Semi-vegetarian	“Eat meat ≤ 1 timeweek”
Australia [21]	Flexitarian	“those who occasionally include conventional meat”
Australia [22]	Flexitarian	“Eating less meat”
Australia [23]	Flexitarian	“…where meat consumption is reduced in some significant way without being eliminated completely.”
Australia [8]	Flexitarian Reducetarian	“…only eat meat on a few days per week or special occasions” and “people who avoid 1 type of meat product but not another (e.g., pescatarian)”
Australia [24]	Semi-vegetarian	Defined as semi-vegetarians “if they excluded red meat”
Belgium [25]	Semi-vegetarian	“people who reduce, but not entirely stop, their meat intake, lean mostly towards health motives.” -Semi-vegetarians: “strongly reduced meat intake” -light semi-vegetarians: “avoid meat one or two days a week”
Belgium [26]	Flexitarian	Flexitarians are…”a growing group of consumers that reduce, but do not ban, meat from their diets.” “Consciously reducing meat intake, but eating meat now and then.”
Belgium [27]	Flexitarian Semi-vegetarian	“Flexitarian: ‘people who consciously reduce their meat intake, but eat meat now and then’“ “Semi-vegetarians: …excluded certain types of meat (red meat, poultry or fish and seafood) from their diet”
Belgium [28]	Flexitarian Semi-vegetarian	“Individuals who have low or occasional meat intake”
Brazil [29]	Flexitarian	“maintain a vegetarian diet most of the time but still eat meat” “avoid the consumption of animal products…not totally eliminating them”
Canada [30]	Semi-vegetarian	“…consumed red meat less than once a month, but included poultry and/or fish in their diets more than once a month.”
Denmark [31]	Flexitarian	“consumes vegetarian food a few days per week”
Europe/USA/Australia/New Zealand/South Africa [32]	Flexitarian	“primarily vegetarian but occasionally eating meat and fish”
Finland [33]	Flexitarian	“abstains from meat occasionally without abandoning meat totally” “a middle category between consumers who regularly eat meat and those who fully abstain from it”
Finland [34]	Flexitarian	Planetary Health Diet: “A flexitarian diet consisting mainly of plant-based foods and containing only small amounts of animal-based foods. It aims to more than double the consumption of fruits, vegetables, legumes, and nuts, and more than half of the global consumption of red meat and added sugars by 2050.”
Finland [35]	Flexitarian	“The main protein sources are as follows per weekly dose: -100 g of red meat -200 g of poultry meat -200 g fish -350 g nuts, -90 g eggs -525 g beans/legumes”
France [36]	Flexitarian	Flexitarian: “limiting meat consumption to a minimum” “Pro-flexitarians”: “I am considering eating meat and fish only very rarely (no more than once a week) and were not flexitarians or vegetarians.”
France [37]	Flexitarian	A consumer study was the result of this study—participants were asked: “which of the following suits you?” 1. I like meat a lot and consume a lot, I can hardly imagine a meal without meat.” = omnivores 2. “I like meat, I consume regularly.” = omnivores 3. I prefer fish to meat, but I eat meat when the opportunity arises.” = omnivores 4. “I try to limit my consumption of meat/fish but I can make exceptions (at the restaurant, at friends’ place)” = flexitarian 5. “I do not eat meat or fish.” = vegetarian 6. “I do not eat meat at all, but fish can be eaten occasionally.” = strong vegetarian trend 7. “I do not eat meat, fish, eggs, or dairy.” = vegan
France [38]	Flexitarian	“diet that limits meat consumption for reasons other than financial. The flexitarian seeks a balanced and varied diet. They therefore also consume animal products”
Germany [39]	Semi-vegetarian	In the study, semi-vegetarians were defined by “those who negated [these] statements but endorsed the statement ‘I mostly eat vegetarian (no red meat but sometimes poultry and/or fish)’“
Germany [40]	Flexitarian	“…broadly characterized by a primarily vegetarian diet pattern with occasional meat or fish consumption. However, a generally accepted definition of flexitarianism does not currently exist.”
Germany [41]	Flexitarian	no description provided
Global [42]	Semi-vegetarian	Review; ~20% of studies allowed participants a semi-vegetarian diet including fish and meat products
Global [43]	Flexitarian Meat reducer Low meat-eaters Semi-vegetarian Casual vegetarians Vegivores	“actively reduce or fully exclude at least some animal products” “cutting back on meat but are not avoiding meat completely” “individuals reporting ‘a lot less’ or ‘slightly less’ consumption v. three years ago for one or more of the four meat types” “do not exceed the maximum meat intake officially recommended by national dietary guidelines” “aim to reduce animal products…but do not strictly exclude any food group” “choose less or without meat meals if they were available” “a predominantly plant-based diet complemented with modest amounts of animal foods”
Global [44]	Flexitarian Flexible vegetarian	“choose mainly plant-based food products…also eat meat and other animal-based products”
Global [45]	Flexitarian	“omnivorous diets that incorporate high amounts of plant-sourced foods; moderate amounts of poultry, dairy and fish; and low amounts of red meat, highly processed foods, and added sugar”
Global [6]	Flexitarian Meat-reducers Semi-vegetarian Demi-vegetarian Flexi-semi-vegetarianism	“…reflects consumers who are ‘meat-reducers,’ eating meat within meals on some but not every day of the week.” -Variability in review studies defined; some restricted intake of red meat, some restricted fish -Adventist health study defined as those consuming dairy products and/or eggs and meat (red meat and poultry1 time/month and < time/week) -”…containing moderate levels of animal products, though [it] was not specified what ‘moderate’ was.” -”…eating red meat, poultry, or fish no more than 1 time/week” -”semi-vegetarian if [they] excluded red meat from [their] diet but ate other meats.”
Global [46]	Flexitarian	“Limit[ing] meat consumption by abstaining from eating meat occasionally.” “…a consumption pattern in which meat is eaten occasionally without avoiding it completely.”
Global [47]	Flexitarian Semi-vegetarian	“…described as a primarily vegetarian diet but allows some animal food consumption, however, the amount and type of animal foods varies, from a specified amount of animal food per month to exclusion of red meat only but inclusion of poultry, fish and other animal foods.” -Includes fruits, vegetables, grains, nuts, seeds, beans, pulses and fish, dairy, eggs, and meat (on some but not all days of the week). Excludes restrictions on meat.
Global [48]	Flexitarian	“…those who eat a mostly vegetarian diet but occasionally eat meat.”
Global [49]	Flexitarian Semi-vegetarian	“The diet consists of the reduction of the consumption of animal products in favor of those plant-based products…”
Global [50]	Flexitarian Semi-vegetarian	“As a sort of semi-vegetarianism, it consists of the eating of a mostly plant-based diet with the occasional inclusion of meat (for instance, during weekends).”
Global [51]	Flexitarian Semi-vegetarian	“meat consumption once or twice a week.” “…are mainly plant-based but occasionally include meat, dairy, and eggs and focus on variety while attempting to minimize animal product consumption.”
Global [52]	Flexitarian	No description provided
Israel [53]	Semi-vegetarian	“Mainly but not completely vegetarian; the consumption of meat or fish less than once weekly and more than once monthly”
Italy [54]	Semi-vegetarian	“…In general, the diet was based mainly on the principles of a Mediterranean diet but with some important modifications: it was semi-vegetarian, with a preference for fish instead of meat, and lower gluten content, than the usual Mediterranean diet.”
Italy [55]	Flexitarian Semi-vegetarian Meat-reducer	“an individual who abstains from eating meat regularly” “exclude at least one type of meat, but not all meats, from their diets”
Italy [56]	Flexitarian	“those who consciously consume a limited quantity of either all types or specific types of meat”
Japan [57]	Semi-vegetarian	-Eggs and milk were used. In other words, our diet was a lacto-ovo-vegetarian diet. -Miso (fermented bean paste) soup, vegetables, fruits, legumes, potatoes, pickled vegetables, and plain yoghurt were served daily. -Fish was served once a week and meat once every 2 weeks, both at about a half the average amount. Patients were provided with several different 4-wk menus on a rotational basis.
Netherlands [58]	Semi-vegetarian	“…a flexitarian diet with 35 g meat/day”
Netherlands [59]	Flexitarian	“Consumers who deliberately reduce meat consumption in frequency.”
Netherlands; Finland [60]	Flexitarian	“abstain from eating meat in certain situations due to varying reasons”
New Zealand [7]	Flexitarian Semi-vegetarian Meat-reducers	“An individual who reduces their consumption of meat and can include those who avoid meat one or two days a week to those who only eat meat on occasion”
New Zealand [61]	Flexitarian	“This group was based on the irregular or non-consumption of white, red and processed meat and was designed to include as many meat restricting groups as possible, such as; ovo-vegetarians, vegetarians, and vegans, and both strict and flexible followers.”
New Zealand [62]	Flexitarian Meat-reducers	“is an individual who limits his or her meat intake yet still includes meat in his or her diet.”
New Zealand [63]	Flexitarian Semi-vegetarian Meat-reducer	“vegetarian diets with moderate amounts of red meat”
New Zealand [64]	Flexitarian Meat-reducers	no description provided
Nigeria [65]	Semi-vegetarian	“semi-vegetarians consuming one to three servings of flesh food per week.”
Norway [66]	Flexitarian Semi-vegetarian	“trying to reduce their intake of animal source food when convenient” “occasionally eat meat”
Poland [67]	Flexitarian Semi-vegetarian	“eating dairy products on regular basis but red meat or poultry at a frequency of at least 1 time per month but less than 1 time per week”
Spain/Europe/Latin America [68]	Flexitarian	“[Flexitarians are] semi-vegetarian because do not exclude meat products (red meat or other meats) but limit their consumption.”
Sweden [69]	Flexitarian	“meat reducers”
Switzerland [70]	Flexitarian Semi-vegetarian	“…flexitarian or semi-vegetarian diet, is a predominantly plant-based diet with the occasional inclusion of meat or fish.”
Switzerland [9]	Flexitarian Semi-vegetarian	Participants in this study were considered flexitarians when they included eggs and dairy products in their daily diet and red meat or poultry at a frequency of ≥1 time/month but ≤1 time/week
Switzerland [71]	Flexitarian Semi-vegetarian	“Mediterranean, Nordic, and flexitarian (semi-vegetarian) diets are omnivorous diets with an emphasis on plant-based foods…”
UK [72]	Semi-vegetarian	“…was characterized by high intakes of meat substitutes, pulses, nuts, and fish.”
UK [73]	Flexitarian	“The definition of a flexitarian diet is variable-it can involve anything from a conscious decision not to eat meat at every meal, to giving up meat once per day per week or eating a primarily vegetarian diet, augmented with the occasional meat burger. Ironically, this means that, at present, the majority of people could be classed as flexitarian, even if they do not necessarily use or welcome that label.”
UK [74]	Flexitarian Meat-reducers	“largely plant-based but with an occasional supplement of meat”
UK [75]	Flexitarian	“eating less meat”
USA [76]	Semi-vegetarian	“…those who avoid red meat but consume fish and poultry” “A person who eats fruits, vegetables, grains, dairy products, eggs, seafood, and chicken but no red meat.”
USA [77]	Semi-vegetarian	“A diet that allowed moderate intake of meat”
USA [78]	Semi-vegetarian	“those who limit meat intake”
USA [12]	Flexitarian Semi-vegetarian	“Cutting back on meat rather than abstaining completely.”
USA [79]	Semi-vegetarian	“limits meat” Contains all foods, including meat, poultry, fish and shellfish, eggs, and dairy, in addition to plant-based foods, such as fruits, vegetables, whole grains, and legumes/beans. However, red meat is limited to 1 time/week; poultry is limited to 5 time/week or less.
USA [80]	Flexitarian	“…consisted of high amounts of fruits, whole-grain cereals, and nuts, low but not null amounts of meat and high amounts of dairy products.”
USA [81]	Flexitarian	“Eating as a semi-vegetarian style”
USA [82]	Flexitarian	“There is a growing need for Americans to shift from a meat-centered diet to a plant-based diet, with meat on the side or in moderation; this is known as a flexitarian diet.”
USA [83]	Flexitarian	“Choosing to reduce meat, dairy, and eggs in favor of more plant-based foods to benefit the environment, improve health, or both.”
USA [84]	Flexitarian	This journal/authors considered flexitarians as those households that “frequently consumed both dairy milk and plant-based beverages.”
USA [85]	Flexitarian Semi-vegetarian	Flexitarian Flip: “dietary shift from the traditional meat-centric diet of Western society to a semi-vegetarian diet”
USA [86]	Semi vegetarian	“Semi vegetarians may consume dairy products and/or eggs, eat some meat (red meat and poultry) ≥1 time/month, and the total of fish and meat ≥1 time/month, but <1 time/week.”
USA [87]	Semi-vegetarian	“Those who ate fish and poultry, but < 1 time/week” Authors defined semi-vegetarians as those “who reported consuming fruits, vegetables, pulses or beans, animal products (chicken or meat, eggs, milk or curd) either daily, weekly, or occasionally, but no fish”
USA/Canada [88]	Semi-vegetarian	“Partially vegetarian diet; consuming meat more often than once a month but less than once per week.”
USA/Canada [89]	Semi-vegetarian	“Semi-vegetarians if intake of red meds, poultry or fish, but not only first was more than or equal to once per month but less than once per week”
USA/Canada [90]	Semi-vegetarian	“Semi-vegetarians were defined as consuming fish at any frequency both consuming other meats <1 time/month or total meat (with red meat and poultry ≥1 time/month and the total of all meats <1 time/week.”
USA/Canada [91]	Semi-vegetarian	“Semi-vegetarians ate red meat, poultry, fish 1 time/month to 1 time/week and eggs or dairy at any level
USA/Canada [92]	Semi-vegetarian	“…ate a total of red meat or poultry ≥1 time/month but all meats combined (including fish) <1 time/week and eggs/dairy in any amount
USA/Canada [93]	Semi-vegetarian	“Consuming dairy products and/or eggs and meat (red meat and poultry ≥ 1 time/month and <1 time/week)”
USA/Canada [94]	Semi-vegetarian	“semi-vegetarians consumed dairy products and/or eggs and (red meat and poultry ≥1 time/month and <1 time/week)”
USA/Canada [95]	Semi-vegetarian	“Semi-vegetarians consumed red meat, poultry and fish less than once per week but more than once per month.”
USA [96]	Flexitarian Semi-vegetarian Meat-reducer	“flexible in the degree to which one consumes meat…there are levels to meat consumption within flexitarianism”
USA [97]	Flexitarian Semi-vegetarian	“consumers who limit their consumption of certain types of meat, such as red meat, poultry, or fish, either in terms of frequency or portion size”
West Africa [98]	Flexitarian	“limitation in consuming meat without being exclusively vegetarian and regardless of financial situation”

**Table 2:** Recommendations for servings of animal-source foods in global food-based dietary guidance (all guidance is provided in daily recommended amounts and applicable to the general adult population ages 19+ unless otherwise specified).

Name of Country/Region [Citation]	Year(s)	Dairy Recommendations for ~2000 kcal Diet	Meat/Poultry/Egg Recommendations for ~2000 kcal Diet	Fish/Seafood Recommendations for ~2000 kcal Diet
Afghanistan [14]	2016	3.5 servings (each serving is ~70 kcal)	2 servings (meat, fish and eggs combined into one group; each serving is ~70 kcal)	
Albania [127]	2008	3 portions (1 portion is 200 mL milk, 150–180 g yogurt, 60–90 g curdle cheese)	1 portion (meat, fish, eggs, and cheese combined into one group; 100–120 g meat or fish, 2–3 eggs, 200 g fresh cheese, 50–60 g ripened cheese)	
Australia [105]	2013	2.5 to 4 serves (1 serve is 1 cup milk, 12 cup evaporated milk, 34 cup yogurt	2 to 2.5 serves (meat, fish, eggs, tofu, nuts and seeds, and legumes/beans in one group; 65 g meat, 80 g poultry, 100 g cooked fish, 2 eggs, 150 g cooked beans/legumes, 170 g tofu, 30 g nuts/seeds)	
Bangladesh [128]	2013	130 g milk or milk product (plain curd) or soya milk	40 g poultry and meat; 30 g eggs	
Barbados [51]	2017	3 mealtime portions per day of “foods from animals” (mealtime portions are equal to an “amount up to the size of the palm or your hand and the thickness of your little finger”)		
Belgium [49]	2019	250 to 500 mL	Maximum 300 g red meat per week; Poultry, eggs, or other meat substitutes 1 to 3 times per week	1–2 times per week (1 time should be oily fish)
Belize [78]	2012	7 portions (defined as portions that give 73–75 kcal) of “foods from animals” daily (meat, fish, milk, eggs, cheese)		
Bulgaria [50]	2006	1 glass milk or yogurt (200 mL) and 50 g cheese	Poultry or lean red meat up to 3 times per week (100 g/serving)	1–2 times per week (150–200 g per serving)
England [52]	2016	No quantitative recommendations provided for adults	No more than 70 g red meat per day	2 times per week (140 g each; 1 time should be oily fish)
Ethiopia [72]	2022	300–400 g	60 g “animal-source foods such as eggs and meat” (fish is depicted as belonging to this food group)	
Fiji [43]	2013	2 meals per day should include “bodybuilding foods” (meat, fish/seafood, poultry, eggs, dairy)		
Geogia [53]	2005	2–3 portions milk, yogurt, sour milk, cheese (250 mL milk; 125 mL yogurt/sour milk; 30 g cheese)	1–3 portions meat, poultry, fish, eggs, legumes (80 g meat, poultry, or fish; 1 egg; 14 cup beans)	
Germany [46]	2024	Daily	No more often than weekly (up to 300 g meat and sausage)	1–2 portions weekly
Ghana [54]	2023	1.5 servings animal source foods (144 g/day; 1 serving = 45–135 g boiled beef or goat, 57–75 g poultry, milk, 67.5–152 g fish, 2 eggs) The variation in serving size reflects different serving size for different types of red meat, poultry, and fish)		
India [136]	2024	300 mL milk or curd	85 g pulses and legumes (30 g can be substituted with fish or meat)	
Ireland [55]	2016	3 servings (200 mL milk or yogurt drink, 124 g yogurt, 25 g cheese)	2 servings of protein foods (50–75 g meat/poultry, 100 g fish, 2 eggs, 34 cup beans, 40 g nuts or seeds)	Up to twice weekly as part of protein foods servings
Israel [56]	2019	Daily	About 2–3 portions chicken/turkey per week; no more than 300 g per week meat	At least 1 portion weekly
Jamaica [73]	2015	5 servings food from animals (75 kcal meat or whole milk or 40 kcal skim milk)		
Japan [58]	2010	2 servings	3–5 servings meat, fish, egg, and soybean dishes	
Kenya [59]	2017	Daily (250 mL milk or yoghurt)	2 times per week (eat 30 g lean meat, fish/seafood, poultry, insects, eggs)	
Lebanon [60]	2013	3 servings (1 cup milk or yogurt, 3 tablespoons powdered milk, 45 g cheese, 8 tablespoons labneh)	5–6.5 servings (1 serving = 30 g meats, 1 egg, 14 cup legumes, 15 g nuts and seeds)	2 servings weekly, at least one from a fatty fish (90 g)
Netherlands [61]	2015	A few portions	Limit red meat consumption	Weekly (preferably fatty fish)
New Zealand [62]	2020	2.5–4 servings (1 cup milk or fortified alternative, 40 g cheese, 200 g yogurt)	2–3 servings legumes, nuts, seeds, fish/seafood, eggs, poultry, red meat (150 g beans/legumes, 170 g tofu, 30 g nuts/seeds, 100 g fish, 2 eggs, 100 g chicken, 65 g meat; less than 500 g cooked meat weekly)	
Nordic [16]	2023	350 mL to 500 mL milk and dairy foods or fortified plant-based alternatives	Less than 350 g red meat per week and minimal intake of poultry	300–450 g weekly (at least 200 g from fatty fish)
Norway [63]	2014	Daily	Less than 500 g meat per week	300–450 g weekly (at least 200 g from fatty fish)
Oman [57]	2024	3 servings (1 cup milk or yogurt, 3 tablespoons powdered milk, 45 g cheese)	5.5 servings fish, poultry, meats, eggs, legumes, nuts and seeds (1 serving = 30 g fish, poultry, or meat, 1 egg, 14 cup legumes, 1 tablespoon peanut butter, 15 g nuts or seeds); no more than 300 g red meat per week	2–3 servings per week as part of protein food intake
Pacific Community [17]	2018	1/6 of the food eaten each day should be “bodybuilding foods” such as fish, lean meat, eggs, beans, and milk (one portion of meat is the palm of your hand)		
Philippines [64]	2012	1 serving (1 glass milk, 12 evaporated milk, 4 tablespoons milk powder)	3–4 servings fish, lean meat, poultry, egg, cheese, dried beans or nuts (1 medium piece fish, 1/3 cup shelled shellfish, 1 egg, 1 slice cheese, 3 cubic centimeters meat/poultry)	
Qatar [65]	2015	Daily (1 cup yogurt or milk, 14 tablespoons labneh, 50 g cheese)	No quantitative guidance listed	Twice weekly
Saint Kitts and Nevis [74]	2010	7 portions (defined as amounts providing 40 to 75 kcal) of “foods from animals” to be eaten daily		
Saint Vincent and the Grenadines [75]	2021	4–8 servings “food from animals” (1 serving = amount equal to 73 kcal; 1 cup milk, 1 oz cheese, 4 oz yogurt, 1 small drumstick, 1 egg, 2 small fish)		
Saudi Arabia [47]	2012	2–3 servings (240 mL milk or laban, 30 g cheese)	2–3 servings meat and legumes (60–90 g meat/poultry/fish/seafood, 12 cup cooked legumes, 1 egg, 4 to 6 tablespoons peanut butter)	
Seychelles [77]	2006	3 portions	Meat/poultry/eggs not mentioned	5 times weekly
Sierra Leone [66]	2016	Daily serving of fish, poultry, meat, milk or eggs		
South Africa [76]	2013	400–500 mL milk, maas, yogurt, cottage cheese, cheese	No more than 560 g red meat weekly; 3–4 eggs weekly	2–3 per week (80–90 g per portion)
Spain [45]	2022	Maximum 3 servings daily (1 serving = 200–250 mL milk, 40–60 g hard cheese, 85–125 g soft cheese, 125 g yogurt)	Maximum 3 servings per week of meat (1 serving = 100–125 g) or up to 4 eggs	3 or more servings per week (1 serving = 125–150 g)
Sri Lanka [44]	2021	12–1 servings (100–200 mL) of milk or fermented milk can be included but not needed daily	1 egg can be eaten daily; Fish recommended daily; 2–4 servings of lean meat or fish can be eaten daily	
Sweden [67]	2015	2–5 dL milk, curdled milk, yogurt	No more than 500 g meat	2–3 times weekly (at least 1 serving fatty fish)
Turkey [137]	2006	2 servings (1 serving = 200 cc milk or yogurt or matchstick size cheese)	Limited amounts of meat; eggs can be a substitute	2 times per week
U.S. [15]	2020–2025	3 cup-equivalents	26 ounce-equivalents (per week)	8 ounce-equivalents (per week)
Zambia [68]	2021	1 serving (245 g milk or yogurt or 1/3 cup cheese)	1 serving poultry, fish, eggs, insects, caterpillars (50–115 g), choosing fish as often as possible	

## Data Availability

The original contributions presented in this study are included in the article/Supplementary Materials. Further inquiries can be directed to the corresponding author.

## Supplementary Materials

The following supporting information can be downloaded at: https://www.mdpi.com/article/10.3390/nu17142369/s1.

## Abbreviations

2020–2025 Dietary Guidelines for Americans (2020 DGA); FBDG (food-based dietary guidance); FAO (Food and Agriculture Organization); IFIC (International Food Information Council); UN (United Nations).
