# Supplementary material for: Vegetarianísh—How “Flexitarian” Eating Patterns Are Defined and Their Role in Global Food-Based Dietary Guidance
Source: Nutrients. 2025 Jul 19;17(14):2369. doi: 10.3390/nu17142369 (PMC12300281; doi:10.3390/nu17142369)
Supplement: Supplementary file 1 [file nutrients-17-02369-s001.zip › nutrients-3685218-supplementary.pdf]

Title: Vegetarianísh- Global Food Based Dietary Guidance That Can Accommodate Flexitarian Eating Patterns

Authors: Hess et al.

Supplementary Information

PubMed search strategy

"flexitarian"

Applied filters: Full text; English

Scopus search strategy

"flexitarian"

Applied filters:

Year: <2024

Subject area: Agriculture and Biological Sciences; Health Professions

Document type: Article

Source type: Journal

Language: English
